# Reducing Stress after Trauma (ReSeT): study protocol for a randomized, controlled trial of an online psychoeducational program and video therapy sessions for children hospitalized after trauma

**DOI:** 10.1186/s13063-023-07806-y

**Published:** 2023-11-28

**Authors:** Heather T. Keenan, Shari L. Wade, Devi Miron, Angela P. Presson, Amy E. Clark, Linda Ewing-Cobbs

**Affiliations:** 1 Department of Pediatrics, University of Utah School of Medicine, 295 Chipeta Way, Salt Lake City, UT 84108 USA; 2 Cincinnati Children’s Hospital Medical Center Division of Pediatric Rehabilitation, Department of Pediatrics, University of Cincinnati College of Medicine, 3333 Burnet Avenue, Cincinnati, OH 45229-3039 USA; 3 Department of Psychiatry and Behavioral Sciences, Tulane University School of Medicine, 1430 Tulane Ave. #8055, New Orleans, LA 70112 USA; 4 Department of Internal Medicine, University of Utah School of Medicine, 30 N Mario Capecchi Dr. , Salt Lake City, UT 84112 USA; 5 Children’s Learning Institute, McGovern Medical School at UTHealth, 7000 Fannin, Suite 2401, Houston, TX 77030 USA

**Keywords:** Pediatric injury, Post-traumatic stress, Trauma-focused cognitive behavioral therapy, Randomized controlled trial

## Abstract

**Background:**

Post-traumatic stress symptoms develop in a quarter to half of injured children affecting their longer-term psychologic and physical health. Evidence-based care exists for post-traumatic stress; however, it is not readily available in some communities. We have developed an eHealth program consisting of online, interactive educational modules and telehealth therapist support based in trauma-focused cognitive behavioral therapy, the Reducing Stress after Trauma (ReSeT) program. We hypothesize that children with post-traumatic stress who participate in ReSeT will have fewer symptoms compared to the usual care control group.

**Methods:**

This is a randomized controlled trial to test the effectiveness of the ReSeT intervention in reducing symptoms of post-traumatic stress compared to a usual care control group. One hundred and six children ages 8–17 years, who were admitted to hospital following an injury, with post-traumatic stress symptoms at 4 weeks post-injury, will be recruited and randomized from the four participating trauma centers. The outcomes compared across groups will be post-traumatic stress symptoms at 10 weeks (primary outcome) controlling for baseline symptoms and at 6 months post-randomization (secondary outcome).

**Discussion:**

ReSeT is an evidence-based program designed to reduce post-traumatic stress symptoms among injured children using an eHealth platform. Currently, the American College of Surgeons standards suggest that trauma programs identify and treat patients at high risk for mental health needs in the trauma system. If effectiveness is demonstrated, ReSeT could help increase access to evidence-based care for children with post-traumatic stress within the trauma system.

**Trial registration:**

ClinicalTrials.gov NCT04838977. 8 April 2021.

## Background

Post-traumatic stress symptoms (PTSS) are associated with significant child and family psychological distress after injury [1–3]. PTSS affects children’s functioning after injury and may include psychological symptoms such as dissociation, avoidance, intrusive thoughts, hyperarousal, and irritability and physical symptoms such as sleep disturbance [4]. Between 25 and 57% of injured children develop significant PTSS [5, 6]. Thus, PTSS following pediatric injury represents a substantial health burden as 3.5 million children sought emergency department care after injury in the USA in 2020 with over 121,994 children under 17 years of age hospitalized for non-fatal injuries [7]. Risk factors for PTSS include pre-injury child adjustment problems such as anxiety, post-injury reactions to the trauma, subjective life threat and fear, low social support and parental PTSS [8–12]. Interestingly, injury severity itself is not associated with development of PTSS symptoms [13]. Because PTSS develops after children are discharged from the hospital, these symptoms are frequently not visible to the trauma system and, thus, may go unrecognized. A study by Newgard and colleagues examining pediatric deaths after admission to the ED for injury showed that self-harm in the year following injury was one of the leading causes of death, underscoring the need for mental health services integrated into the trauma system [14]. Thus, a screening method and a brief, effective treatment that could be delivered through the trauma system has the potential to fill a needed gap in therapy.

Evidence-based therapies for PTSS do exist. Cognitive behavioral therapy (CBT) is an evidence-based treatment that has shown greater improvement than supportive child-centered therapies for childhood anxiety and behavioral problems in general and for children with PTSS in particular [15, 16]. Evidence-based treatments such as CBT [17–20] typically include education about emotions, identifying and modifying negative thoughts and appraisals, and training in coping skills and relaxation and may include trauma exposure to reduce anxiety and fear (e.g., graded exposure, reducing avoidance) [18, 19]. Trauma-focused CBT was developed to intervene following a range of specific traumas, including interpersonal violence, accidents and injuries, or exposure to/witnessing events occurring to others [17, 21]. Trauma-focused CBT addresses trauma impact using components of CBT and recounting of the trauma narrative to address cognitive distortions and provide exposure to the child’s personal trauma experience. As children retell their experience, they apply the CBT skills that they have learned to restructure and master the response to their memories [18]. The trauma narrative provides children with tools to engage with trauma memory, organize and articulate a positive interpretation of their trauma story, and modify basic core beliefs about the world [21]. Community access to these evidence-based treatments for PTSS is limited in many regions due to families’ lack of health insurance coverage and a paucity of providers especially in more rural areas [22].

eHealth approaches may improve access and reduce barriers to care. eHealth delivers psychological therapy to children and adults with a variety of conditions including PTSD, anxiety, and traumatic brain injury with comparable efficacy to face-to-face approaches [23]. Online alone preventive treatment for PTSS has been successful in a small pilot [10]; however, therapist involvement for treatment may be associated with larger treatment effects [24]. eHealth approaches may also improve access to care especially in rural communities and reduce barriers including the stigma sometimes associated with seeking mental health care [25]. Translating existing PTSS treatments to a therapist-involved eHealth delivery system may substantially improve access to and participation in treatment without reducing efficacy. Many trauma systems, especially those with large rural catchment areas, have active telehealth programs into which an eHealth therapy program could be integrated [26], thereby improving widespread dissemination and implementation [27].

We have developed an online psychoeducational program that includes web-based psychoeducation about PTSS and elements of CBT (e.g., stress management strategies, reframing unhelpful thoughts) with sharing the trauma narrative and graded exposures to the most distressing memories of the experience. The online content is accompanied by weekly meetings with a therapist via videoconference. If successful, these methods could be integrated into trauma systems with existing telehealth programs, providing a systems-level intervention.

### Objectives

The goal of the current randomized controlled trial is to evaluate the Reducing Trauma After Stress (ReSeT) program. ReSeT is a therapist-involved online intervention that incorporates both elements of CBT and graded exposures for children with post-traumatic stress who have been hospitalized following an injury. ReSeT provides online education about post-traumatic stress, identifying and managing feelings, and teaches coping skills which are then used to process the exposure through the trauma narrative. Support is provided by a therapist in weekly online meetings. The goal is to improve children’s psychological outcome after a traumatic injury and give them tools to manage future challenges.

### Aims and hypotheses

This study was designed to test the efficacy of ReSeT to reduce PTSS after hospitalization for an injury compared to usual care among children who have elevated symptoms of PTSS at 4 weeks post-injury. Our first aim is to compare the groups on the outcome of PTSS at 10 weeks and 6 months post-randomization as measured by the Child PTSD Scale (CPSS) [28]. Second, we will explore whether child pre-existing psychological health modifies outcomes. Third, we will examine whether there is heterogeneity of treatment effects among subgroups including those with a higher initial symptom burden compared to those with a lower symptom burden, age, and sex.

We hypothesize that children who receive the ReSeT intervention will have a clinically important and statistically significant reduction in symptoms at 10 weeks post-randomization (primary outcome), and this effect will be shown to be durable at 6 months. Finally, we expect that we will find heterogeneity of treatment effect among subgroups of children including those with a higher symptom burden, and those with higher levels of pre-existing psychological problems and with lower functioning families.

### Study design

This is a multicentered randomized controlled trial with a 1:1 assignment of the intervention (ReSeT) to control (usual care) that aims to evaluate whether ReSeT is superior to usual care in reducing children’s symptom levels. The intervention lasts 8 weeks. Outcome assessment occurs 1-week post- injury (baseline or pre-injury measure), with assessment for trial entry (elevated stress) at 4 weeks post-injury. The primary study outcome is assessed at the completion of the intervention (10 weeks post-randomization) and durability of effect is assessed at 6 months post-randomization. This study was registered at ClinicalTrials.gov (NCT04838977) 8 April 2021. Study investigators are handling all aspects of trial management including training of research coordinators, monitoring enrollment, and checking data quality. Investigators meet bi-weekly to review subject accrual, monitor processes, and discuss any potential needed protocol modifications. Data are managed by the Utah Data Coordinating Center at the University of Utah who developed and maintain the REDCap database.

### Setting

Children are recruited from four level 1 pediatric trauma centers in the USA: Cincinnati Children’s Hospital and Medical Center (Cincinnati, Ohio); Children’s Memorial Hermann Hospital (Houston, Texas); Nationwide Children’s Hospital (Columbus, Ohio); and, Primary Children’s Hospital (Salt Lake City, Utah). Level 1 trauma centers are regional referral hospitals that are capable of providing all aspects of trauma care from prevention through rehabilitation. Children are recruited from the inpatient wards including the pediatric intensive care unit, the surgical unit, and the short stay units (< 23 h hospital admissions).

### Participant eligibility, recruitment, enrollment and randomization

#### Eligibility

Children aged 8 to 17 years hospitalized for trauma are eligible for the study. To be included, children and at least one parent must speak English and have broadband internet availability at their home address. Children with broadband availability but without an internet subscription or computer will be provided with a tablet and internet subscription. Children may not have moderate or severe TBI, as defined by a Glasgow Coma Score of < 13 as these children may find it difficult to participate in therapy. Exclusionary criteria include pre-existing severe psychiatric problems requiring hospitalization; developmental disorders which would preclude participation in therapy; children injured by abuse or through interpersonal violence or a self-inflicted injury; children currently receiving psychotherapy; children hospitalized for over 30 days; and children injured in an event where there was a death of a family member or friend.

#### Recruitment and enrolment

Research coordinators screen all hospital trauma admissions daily using the electronic medical record. Families are then approached in person or by telephone to confirm eligibility and request verbal consent to participate in the study. Families who are missed are contacted by postal mail to inform them about the study. Families who agree to participate are sent a link to a REDCap (Research Electronic Data Capture) website 1 week following injury and they are asked to fill in baseline information including demographics and study outcomes to obtain baseline measures. Study measures are shown in Table 1.

**Table 1:** Schedule of enrolment, interventions and assessments

Timepoint		Study period			
		**Eligibility and enrolment**	**Informed consent and allocation**	**Post-allocation**	
		**Baseline** **1 week post-injury (− *t***_**1**_**)** **1 week post-injury**	**4 weeks post-injury (*t***_**0**_**)** **1 month post-injury**	**10 weeks (*t***_***1***_**)**	**6 months (*t***_***2***_**)**
**Intervention: ReSeT program or Usual Care**					
**Assessments**^a^					
	***Child Psychological Health***				
**Primary Outcome**	Child PTSD Scale (CPSS) [28]	**B**	**B**	**C**	**B**
**Comorbid Psychological Health and Coping**	Children’s Post-Traumatic Cognitions Inventory (CPTCI) SF [35, 36]	**C**			
	Screen Child Anxiety and Related Emotional Disorders (SCARED) [37]	**C**			
	PROMIS Scales – Anger, Anxiety, Depression, Psychological Stress, Physical Stress SF [38–40]	**B**			**B**
	Connor-Davidson Short Form [41]	**C**			
	Traumatic Events Screening Inventory (TESI) [42]	**C**			
	COVID-19 Life Impact (Child)	**C**			
	Vanderbilt ADHD Diagnostic Rating Scale (VADRS) [43]	**P**			
	***Child Physical Health***				
	PROMIS Scales: Global Health [44]	**B**		**B**	**B**
	PROMIS Scale – Sleep [45]	**P**		**P**	**P**
	***Child Health-Related Quality of Life***				
	PedsQL4.0 [46]	**P**		**P**	**P**
	***Family Function/Parent Psychological Health***				
**Family Function**	McMaster Family assessment [47]	**P**			
**Parent Psychological Health**	PROMIS Profile [48]	**P**			
	PTSD Checklist-DSM 5 [49]	**P**			
	***End of treatment surveys***				
	Satisfaction survey			B	

^a^*C *Child report, *P *Parent report, *B *Both parent and child

**Fig. 1:**
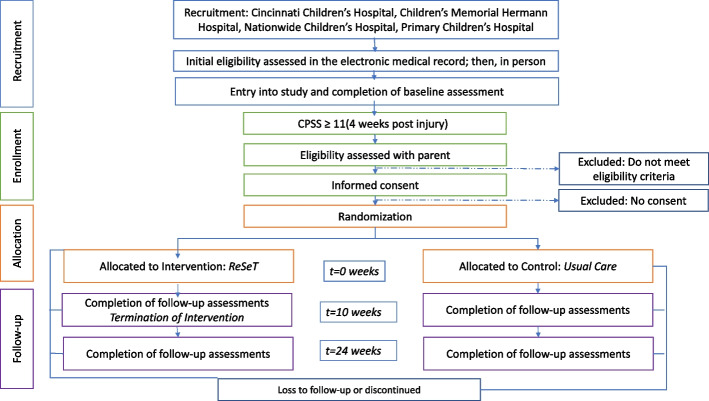
ReSeT flow chart from recruitment through completion

Parents and children are asked to complete a measure of child PTSS, the Child PTSD Scale (CPSS), at 1 week and 4 weeks post-injury [28]. The CPSS was selected as it is a well-established open source questionnaire with favorable sensitivity and specific for identifying PTSS and predicting children’s response to treatment that has been used previously in similar populations [10, 29]. To assess the child’s stress symptoms, the highest value of the parent or child’s response to each item on the CPSS are summed. If the sum is greater or equal to 11 at 4 weeks post-injury, the child is eligible for randomization. The summed value is used as parents may be unaware of children’s internalizing symptoms and children may self-report low values on some items due to avoidance as suggested by Mai and Scheeringa [30]. Families whose child has a CPSS ≥ 11 meet with the research coordinator for their site via videoconference to recheck eligibility requirements and to review the full trial information. After time to ask any remaining questions or consult with other family members, parents are asked to complete informed consent via a REDCap module and children are asked for assent. After informed consent and assent, the family is enrolled in the trial and proceeds to randomization. There are no additional consent provisions. Figure 1 shows the consort diagram from recruitment through follow-up.

#### Randomization

Randomization occurs at the time of the 4-week CPSS. One hundred and six children will be randomized from the participating centers using a computer-generated random assignment sequence prepared by the study statistician and uploaded into the REDCap randomization module*.* Stratification will occur by site and by age group (8–11 years, 12–17 years). Randomization is conducted while the coordinator is speaking with the family via videoconference in order to enable a “warm handoff” to the therapist for families randomized to the treatment group. The randomization sequence is not visible to the research coordinators.

### Intervention

The ReSeT intervention includes 8 online psychoeducational sessions with each session followed by a meeting with a post-doctoral psychology fellow or a doctorate level psychology student supervised by a psychologist with expertise in trauma intervention. The psychoeducational content is delivered online from a dedicated website that has a logon for each individual. Each of the 8 sessions has 3–4 short interactive videos to help children learn the concepts being taught. Concepts are reinforced with brief homework assignments. The therapist meetings are conducted via HIPAA compliant video conferencing. Parents are asked to attend therapy sessions with children younger than age 11 years and may meet the therapist before and after the session for older children as outcomes have been shown to improve with parent involvement [31].

In the first session, the therapist goes through the online video content with the child and parent to teach them about the program and trouble shoot problems that families may have with the technology. After this initial visit, the next three sessions focus on aspects of cognitive behavioral therapy including understanding feelings, identifying helpful and unhelpful thoughts, understanding that thoughts can change outcomes, and cognitive reframing. Videos for each session engage children in learning the concepts and learning skills that they are then able to practice with the therapist and at home including belly breathing, positive imagery, cognitive reframing, and using feelings thermometers to rate the intensity of feelings and the effectiveness of stress management therapies. Videos include interactive content to engage children. Children may go to the ReSeT website to practice skills using the videos as often as they wish and are asked to rate their feelings with the feeling thermometer on the website. Sessions 5 to 7 involve telling the most stressful or scary aspects of the trauma narrative while practicing stress management techniques to allow desensitization to the trauma. The video content shows a child who was injured sharing her trauma narrative with the therapist. The videos show the child creating a hierarchy of least scary to most scary events after injury, and working through the hierarchy with therapist by using the skills that were taught in the prior sessions if she becomes stressed. In the session with the therapist, children build their own trauma narrative hierarchy by identifying and ranking the least to most scary aspects of the trauma and practicing their skills while they talk about what happened. The final session involves a wrap up and resilience plan in which children learn that stress may return and make a plan to manage it. If the parent requests or the therapist feels that the child needs ongoing care after the intervention is completed, appropriate referrals are made by the PI at each site.

Psychoeducational resources for parents are available on the website and include education about self-care for the parent, managing their own stress symptoms, positive parenting and managing child behavior, and managing sleep problems for children. Parents view these resources independently and their completion is not required.

Control subjects will receive usual care. All aspects of care are permitted during the trial, including psychological support. Families are asked whether psychological support was received outside of the ReSeT program at the 10-week and 6-month outcome assessments. Usual care was selected as the comparator as it reflects what children currently receive in the trauma system. Families who request a referral for psychological resources either during or after the completion of the study are given a referral by the site investigator.

#### Criteria for discontinuing the intervention

Participants are free to discontinue the intervention at any time; however, modification will not be made to the intervention.

#### Strategies to improve adherence

Participants are reminded to complete measures using automated REDCap reminders and text messages from the research coordinators. Participants receive a small monetary incentive for completion of trial activities.

#### Relevant concomitant care

While we exclude children from the trial if they are currently receiving therapy so as not to interfere with their ongoing care, all types of concomitant care are allowed during the trial including seeking therapy outside of the trial.

#### Data management and security

We use REDCap to support this study. REDCap is a secure, web-based, data capture tool, hosted at the University of Utah, which allows participant outcomes to be directly entered by participants. Participants and their parents enter all baseline information including private identifiers and subsequent assessments into REDCap. Study personnel enter medical information from the patients’ charts into the REDCap database. Study personnel may only view participants from their own site. REDCap is programmed with plausible ranges for entered values. The ReSeT website, where participants view the videos, is encrypted and password protected. Each family is given a unique logon and create a password. Each participant is assigned a study ID that is linked to the REDCap database and the ReSeT website. At the time of analysis, an analysis file will be created that merges de-identified information from the ReSeT website and REDCap database by study ID. Data checks are run prior to each DSMB meeting and site research coordinators are asked to source verify any missing variables; however, participants may skip questions in the assessments if they do not wish to respond. The REDCap instance is HIPAA compliant and is housed at the University of Utah’s secure, Federal Information Security Management Act compliant, Data Coordinating Center behind University firewalls.

### Quality control measures

#### Training research assistants

Research assistants are trained on the study protocol. Training includes (1) review of the ReSeT manual; (2) use of the REDCap; (3) best practices for communications with families including how to approach families in person, by phone or by text; (4) consent procedures including practice of providing informed consent and using the REDCap consent module. Training materials are kept with updated versions stored in a Box folder accessible to all sites.

#### Supervision of therapists

Therapists are trained in the use of the website and delivery of therapy. They are trained through reading and discussion of the training manual, viewing video trainings, and discussions with a licensed clinical psychologist. All cases are supervised weekly by two experienced clinical psychologists who helped to develop the intervention. During supervision meetings, each aspect of the completed sessions is discussed and future sessions are planned to ensure fidelity to the manual.

#### Masking

This is an unblinded study as it is not feasible to blind the research coordinators, the therapists, or the participants. Participants in the treatment and control groups complete outcome measures by directly entering responses into REDCap and the analysis file is de-identified which reduces the risk of bias from unblinded study personnel. Because this study is not blinded, there are no procedures for unblinding.

#### Fidelity to treatment

Therapists complete a detailed fidelity checklist after each session to document that each element in the treatment manual for that session is completed. Additional documentation includes the total duration of the session, who attended, and time spent with the participant and the participant’s parent. The therapist also rates the participant’s engagement in the session using a Likert scale.

#### Baseline measures

Parents will be asked to complete family demographic measures including family composition, self-identified race and ethnicity, health insurance status, and income. Parents will be asked to complete measures of family functioning and parental psychological health as these may directly impact children’s recovery [32, 33]. Children complete baseline mental health questionnaires. Both children and parents separately complete a measure of post-traumatic stress reflecting the 1-week post-injury timepoint. Research coordinators will abstract injury information from the chart to include injury mechanism, injury severity score, admission unit and whether there was an operative intervention, and length of stay using a structured format. Abbreviated injury scores will be provided by each hospital’s trauma registrar [34].

#### Outcome evaluation

All child participants and parents will be asked to complete outcome evaluations through REDCap at 10 weeks and 6 months post-randomization. Participants randomized to the intervention will be asked to complete outcome evaluations regardless of whether they completed all 8 sessions. Table 1 displays schedule of enrolment, interventions and assessments with their timing for participants and their parents. Measures include child physical and psychological health as well as brief measures of family function and parent psychological health as these may affect either children’s likelihood of having PTSS or their response to treatment.

## Study outcomes

### Primary outcome

#### The child PTSD scale (CPSS)

The CPSS is a validated child report and parent proxy measure of PTSS [28]. The measure is comprised of 24 items. The CPSS includes 17 questions mapping on to DSM-IV symptoms of PTSD rated on a 4-point scale that indicates how frequently each symptom occurs (0 = not at all, 1 = once a week or less/once in a while, 2 = 2 to 4 times a week/half the time, 3 = 5 or more times a week/almost always). In addition, the CPSS includes seven questions regarding functional impairment. These questions are scored dichotomously as absent (0) or present (1). Scores range from 0 to 7, with higher scores indicating greater functional impairment. A composite of the parent and child CPSS will be used for the primary outcome. We will sum the highest score on each item from the parent and the child as suggested by Mai and Scheeringa for entry into the trial and as the primary outcome [30].

### Secondary outcome

The secondary outcome is the composite CPSS assessed at 6 months post-randomization that will assess the durability of any treatment effect.

## Potential moderators of treatment outcome: child measures

### Screen child anxiety and related emotional disorders (SCARED)

SCARED is a questionnaire that screens for anxiety and emotional disorders in children [37]. The child self-report version will be used in this study, which consists of 41 items rated on a 3-point Likert scale (0 = *not true or hardly ever true*, 1 = *somewhat true or sometimes true*, 2 = *very true or often true*). This measure yields raw scores for anxiety disorder, panic disorder, generalized anxiety disorder, separation anxiety, social anxiety disorder, and significant school avoidance.

### Patient-reported outcomes measurement information system (PROMIS)

PROMIS is a National Institutes of Health (NIH) initiative created to advance assessment of patient-reported outcomes [44]. We will use both the pediatric self-report and parent proxy short forms for the anger, anxiety, depression, psychological stress, and physical stress scales which are each eight items and the global health scale which is seven items. Additionally, the eight-item sleep disturbance questionnaire will be completed by parents.

### The pediatric quality of life inventory (PedsQL 4.0)

The PedsQL is a well-validated parent-report measure consisting of 23 items that takes less than 5 min to complete [46]. The PedsQL evaluates health-related quality of life by assessing the dimensions of physical, emotional, social, and cognitive functioning in children from infancy to 18 years of life age.

### Connor-Davidson resiliency scale (CD-RISC)

The CD-RISC is a self-report questionnaire to measure resilience [41]. The shorter version will be used in this study, which consist of 10 items rated on a 4-point Likert scale (0 = *rarely true*, 1 = *sometimes true*, 2 = *often true*, 3 = *true nearly all of the time*). The 10-item version has demonstrated good internal consistency and construct validity.

### Traumatic events screening inventory – child report form revised (TESI-CRF-R)

The TESI-CRF-R is a well-validated measure consisting of 24 items to assess traumatic experiences among youth ages 6 to 18 years [42]. The TESI-CRF-R assess a child’s experience of a variety of potential traumatic events including current and previous injuries, hospitalizations, domestic violence, community violence, disasters, accidents, physical abuse, and sexual abuse.

### Vanderbilt ADHD diagnostic rating scale (VADRS)

The VADRS is a parent-report scale with good internal consistency, factor structure, and concurrent validity for the assessment of attention-deficit hyperactivity disorder (ADHD) and externalizing difficulties [43]. We will be administering 40 items of the VADRS including the ADHD scale (18 items), ODD scale (8 items), and conduct disorder scale (14 items). The VADRS includes the 18 *DSM-IV* ADHD symptoms rated on a 4-point scale that indicates how frequently each ADHD symptom occurs (0 = *never*, 1 = *occasionally*, 2 = *often*, 3 = *very often*). In addition, the VADRS includes ODD and conduct disorder scales that are reliable.

### Child post-traumatic cognitions inventory (CPTCI) S

The CPTCI-SF is a self-report measure of negative post-trauma cognitions [35, 36]. The CPTCI-S consists of 10 questions for self-report asking about reactions after a frightening event. Each question is ranked on a 4-point Likert scale.

## Potential moderators of treatment outcome: parent and family measures

### Family assessment device-general functioning scale (FAD-GF)

The FAD-GF is a 12-item sub-scale of the FAD that has demonstrated reliability and validity in assessing global family function [47].

### PROMIS adult self-report

This 29-item parent self-report assesses each of the seven PROMIS domains (physical function, fatigue, pain interference, depressive symptoms, anxiety, ability to participate in social roles and activities, and sleep disturbance) with 4 questions [48]. Each of the questions is ranked on a 5-point Likert scale. The last item is an 11-point rating scale that assesses pain intensity.

### PTSD Checklist-DSM5 (PCL-5)

The PCL-5 is a 20-item parent self-report measure that assesses the 20 DSM-5 symptoms of PTSD [49]. Respondents rate the frequency of their PTSD symptoms over the past 2 weeks on a 4-point Likert scale (0 = *not at all or only one* time to 3 = *5 or more times a week/almost* always). A total symptom severity score can be obtained by summing the scores for each of the items.

### COVID exposure and family impact survey (CEFIS)

CEFIS is a caregiver report measure developed in March/April 2020 to measure potentially traumatic aspects of the COVID-19 epidemic as they may influence pediatric study outcomes. It consists of 25 items related to COVID-19-related events and 12 items that measure their impact. The CEFIS was registered and made available on April 22, 2020, with the National Institutes of Health Disaster Information Management Research Center. The scale was validated in 2021 and has excellent internal reliability [50].

### Satisfaction surveys

Both parents and children complete brief surveys asking about their overall satisfaction with the program and whether they liked or disliked specific aspects of the program. These surveys, developed by the investigators, are for potential future modifications of the program.

## Data analysis

The primary analysis is an intention to treat analysis including all patients who are randomized regardless of their adherence. We will also do an analysis of per-protocol efficacy of participants who received at least 4 of the 8 sessions and completed the 10-week post-randomization CPSS. All analysis will be performed using SAS® Software version 9.4 or later whenever possible. Other software packages, including R, STATA and StatXact®, may be used for particular specialized procedures.

Demographic variables will be summarized with descriptive statistics. Parent and child psychological health scales and subscales will be scored and described. Data will be examined for missingness and outliers. We will examine whether there are baseline differences between groups in the demographic or health scales in case randomization did not adequately balance the groups.

The purpose of the primary analysis is to test the null hypothesis that here is no difference in mean 10-week CPSS score between the CBT and usual care arms. The alternate hypothesis is that there is a difference in mean 10-week CPSS score between the CBT and usual care arms. To test our hypothesis, we will use a generalized linear regression model with an outcome of 10-week CPSS score. Generalized linear regression was chosen as many survey outcomes can be heavily skewed; this approach is flexible enough to accommodate outcomes with either a normal or non-normal distribution [51]. Covariates in the model will include an indicator for treatment group, baseline (1 month) CPSS score, time since randomization, site, and age group. The coefficient for treatment group will answer our primary efficacy question regarding whether 10-week CPSS score differs between CBT versus usual care arms. Adjustment for the baseline CPSS score provides the structure of an analysis of covariance (ANCOVA) to improve statistical power [52]. Time since randomization will be included as a continuous variable to control for a variability among timing of when patients complete the outcome in the event that these differ by group. Site and age group will be included since they were stratification variables used in the randomization protocol.

Regression diagnostics will include plotting residuals vs fitted values to assess linearity, the square root of the standardized residuals vs fitted values (i.e., “Scale-Location”) to assess variance homogeneity, qq plot to assess normality, and standardized residual vs leverage to assess for outliers. The choice of outcome model will depend on the distribution of the CPSS score, which will be visualized using a histogram and density curves stratified by treatment arm. Provided the CPSS score distribution is sufficiently symmetric, we will use a normal outcome model. If the regression assumptions are not reasonably met, we will consider alternative models such as gamma or lognormal. As a sensitivity analysis, we will repeat the above model excluding time since baseline, and also excluding any CBT subjects that had a CPSS score collected after 12 weeks.

If there is an issue with treatment adherence, we will use an instrumental variables approach to estimate treatment effects under different treatment doses. The advantage of this approach over a per-protocol analysis is that it enables us to analyze patients according to their randomized assignment.

## Analysis of secondary outcome

For analysis of the 6-month CPSS time point, which will assess persistence of the treatment effect between the CBT and usual care groups, we will use restricted maximum likelihood estimation [53] under a generalized linear mixed model. We will again adjust for the same variables as described above for the main efficacy analysis, except that the outcome timepoint also will be included as binary (10 weeks vs 6 months). The primary efficacy evaluation will be performed using an interaction between the CBT versus usual care group indicator and time. We will adjust for 1 month baseline CPSS in this analysis.

## Analysis of exploratory outcomes 

Exploratory outcomes will examine whether subgroups differ in their response to treatment. Subgroups will include whether separate child and parent CPSS scores instead of the combined scores alters the treatment effect, whether children with a higher initial CPSS (CPSS ≥ 15) differ from those with a lower CPSS, and whether children with pre-existing depression or anxiety respond differently to treatment. Additionally, we will explore whether treatment differences are seen by age or sex. As the non-CPSS exploratory outcomes are not assessed at the 1 month time point, but they are available at 1 week after injury, we will use this 1 week time point as our baseline measure.

### Missing data

Multiple imputation will be implemented for our intention to treat analysis if there is notable missingness in the 10-week CPSS, or an imbalance in missingness between treatment arms [54]. Multiple imputation is particularly useful if there are auxiliary variables that are highly correlated with both the study outcomes and risk of missingness. We will include the following auxiliary variables: child and parent measures (Table 1) and patient demographic factors. In the event that multiple imputation is used, we will generate 20 imputed data sets which contain imputed values of the primary CPSS outcome (if R is used, we will use the mice package). The imputation model will include the outcomes and all predictor variables including the randomized treatment assignment, auxiliary variables, and the baseline level of CPSS.

### Power analysis and sample size

The power analysis was conducted in PASS v. 16.0.6. We assumed a correlation between CPSS at trial entry and the 10-week post-intervention CPSS of 0.55 based on prior work by the study team. We estimated a conservative effect size of 0.5 based on Kassam-Adams’ pilot of an online only PTSS preventive treatment among injured children [10]. Based on these assumptions, using an ANCOVA model, we estimate that we would have 80% power at a 0.05 significance level to detect this difference with 45 participants per group (90 total). We expect a loss to follow-up rate of up to 15%; thus, we need to enroll 53 participants per group for 106 total.

## Data safety and monitoring board

A Data Safety and Monitoring Board (DSMB) will meet every 6 months via video conference to monitor the trial for accrual and retention, confidentiality of the study data, safety of participants, performance of study sites, and other factors that may affect study outcome. The DSMB consists of two psychologists who are skilled in the treatment of children with post-traumatic stress, and a statistician who is skilled in clinical trials. The DSMB is independent from the sponsor and has declared that no competing interests exist. At the first DSMB meeting, members will review the study protocol and data collection procedures. At subsequent meetings, issues discussed will include the conduct and progress of the study, including patient recruitment, data quality, general adherence, compliance with protocol, any protocol modifications, and any other logistical matters that may affect either the conduct or outcome of the study. No interim statistical analyses are planned for the trial; however, the DSMB will have access to subject accrual and aggregated data by study group at each meeting. The DSMB charter is maintained in a Box account at the University of Utah. The DSMB will provide a report after each meeting to summarize their findings and make recommendations concerning continuation, termination, or other modifications to the study.

### Adverse events

The study team will collect any adverse events. All treatment and control patients have access to the study coordinator text numbers and treatment patients meet with the therapist. Any participant needing additional support will be provided referrals by the study team. Adverse events will be reported to the Institutional Review Board and to the DSMB. The DSMB will monitor the occurrence of adverse events. Serious adverse events will be reported to the DSMB within 7 days of site awareness.

### Protocol amendments

The need for protocol amendments will be discussed among the investigators. If all investigators approve the amendments, the requested change will be submitted to the Institutional Review Board, the DSMB, and the National Clinical Trials website.

## Dissemination policy

The investigators will have access to the all data, perform the main analyses and publish them in the peer-reviewed literature and present them at relevant meetings. We do not plan to use professional writers. Study results will be available in ClinicalTrials.gov. Families will be notified of trial results through an emailed newsletter at the trial conclusion.

## Discussion

This study evaluates whether the use of an eHealth educational program combined with telehealth visits with a therapist is effective to treat children with PTSS after admission to hospital following an injury. The study builds on prior work that demonstrates that TF-CBT is effective in decreasing stress symptoms and that eHealth methods work similarly to in-person counseling for children with depression and anxiety [18, 55]. Because injury severity is not a predictor for the development of post-traumatic stress symptoms [13], the population at risk is potentially much larger, extending to children who are treated and released from the emergency department following injury.

The current study focuses on the reduction of stress symptoms; however, it offers an opportunity to examine the effects of the trial on other outcomes including health-related quality of life and symptoms such as anxiety and depression. It also allows the exploration of the interaction of parents’ mental health symptoms and the family environment with children’s symptoms [56]. This may provide other targets for intervention.

If this trial is successful, it could be scaled for use within the pediatric trauma system as many trauma systems have existing telehealth capabilities designed to reach their large catchment areas. Recently, identification and treatment of patients at high risk for mental health needs within the trauma system was recommended by the American College of Surgeons as a Type II standard [57]. An eHealth program, such as ReSeT, would be especially useful for trauma programs with large rural catchments where referrals to evidence-based treatments may not be feasible. If the trial is successful, ReSeT could be extended to use these for other causes of pediatric post-traumatic stress such as natural disasters.

## Trial status

Trial enrollment was started on 23 July 2021 and will be completed as of 30 November 2023. Follow-up of all study subjects will be complete as of 15 June 2024. Protocol version #3; date 3 August 2023 [3].

## Data Availability

A fully de-identified data (direct and indirect identifiers) containing the data that this study relies upon will be available on request 3 years after the completion of all study procedures with accompanying code for the main analysis. Because these data contain clinical information, any data or resources shared upon request will be released only after documentation (e.g., data sharing agreements) as appropriate is completed by the relevant entities. Users must agree to the conditions of use governing access to the public release data, including restrictions against attempting to identify study participants, destruction of the data after analyses are completed, reporting responsibilities, restrictions on redistribution of the data to third parties, proper acknowledgement of the data resource, information provided to users will not be used for commercial purposes, and will not be redistributed to third parties.

## Abbreviations

CBT: Cognitive behavioral therapy
CD-RISC: Connor-davidson resiliency scale
CPTCI-SF: Child post-traumatic cognitions inventory short form
CPSS: Child PTSD scale
DSMB: Data safety and monitoring
FAD-GF: Family assessment device-general functioning scale
PedsQL: Pediatric quality of life inventory
PCL-5: PTSD Checklist-DSM5
PROMIS: Patient-reported outcomes measurement information system
PTSD: Post-traumatic stress disorder
PTSS: Post-traumatic stress symptoms
REDCap: Research electronic data capture
SCARED: Screen child anxiety and related emotional disorders
TESI-CRF-R: Traumatic events screening inventory – child report form revised
VADRS: Vanderbilt ADHD diagnostic rating scale
